# Nutrient Deprivation in *Artemia franciscana*: Developmental Stage, Nutritional History, and Phenotypes Linked to Conserved Pathways

**DOI:** 10.3390/ijms27083621

**Published:** 2026-04-18

**Authors:** Nikola Mitovic, Milena Maya Stamatoski, Dragan Ilic, Dalia Yassin Makki, Hala Alsaadi, Darko Puflovic, Milica Milosevic, Mirjana Jovanovic, Maja Milosevic Nale, Draško Gostiljac

**Affiliations:** 1 Department of Pathophysiology, Faculty of Medicine, University of Belgrade, 11000 Belgrade, Serbia; milenastama@gmail.com (M.M.S.); ilicd736@gmail.com (D.I.); daliaymakki@gmail.com (D.Y.M.); alsaadih09@gmail.com (H.A.); mirjana.jovanovic@med.bg.ac.rs (M.J.); 2 Faculty of Electronic Engineering, University of Niš, 18000 Niš, Serbia; darko.puflovic@elfak.ni.ac.rs; 3 Institute for Cardiovascular Diseases “Dedinje”, 11040 Belgrade, Serbia; milosevic.a.milica13@gmail.com (M.M.); maja.a.milosevic@gmail.com (M.M.N.); 4 Faculty of Medicine, University of Belgrade, 11000 Belgrade, Serbia; 5 Clinic for Endocrinology, Diabetes and Metabolic Diseases, 11000 Belgrade, Serbia

**Keywords:** *Artemia franciscana*, starvation, nutrient deprivation, locomotor activity, morphometric analysis, developmental stage, invertebrate model

## Abstract

Starvation is a fundamental physiological stressor that triggers conserved adaptive responses across species, however, its effects are shaped by both developmental stage and prior nutritional history. This study aimed to investigate the effects of acute nutrient deprivation in *Artemia franciscana*, comparing newly hatched nauplii and adult individuals previously exposed to reduced caloric intake during development. Organisms were subjected to starvation for 24, 48, and 72 h, and mortality, morphometric parameters, and locomotor activity were assessed, complemented by in silico analysis of starvation-related pathways. Starvation induced distinct responses between groups, with markedly higher mortality in adults compared to nauplii. While these differences reflect developmental stage-associated responses, they are also influenced by prior nutritional history. Body length was significantly reduced under starvation in both developmental stages, while antennal length remained largely unchanged. Locomotor activity, including distance travelled and swimming velocity, was consistently decreased, indicating energy-conserving behavioral adaptation. Partial recovery of locomotor performance and antennal length was observed following restoration of feeding. Bioinformatic analysis suggested the presence of conserved autophagy-related genes and enrichment of pathways associated with autophagy and *TOR* signaling. However, these findings should be interpreted as hypothesis-generating, given the reliance on a proxy species for pathway inference. These findings indicate that starvation responses in *A. franciscana* are shaped by an interaction between developmental stage and prior nutritional history, supported by conserved stress–response pathways, highlighting the potential of this model for studying metabolic stress responses.

## 1. Introduction

Starvation and nutrient deprivation represent fundamental physiological stressors encountered by organisms that belong to different branches of the tree of life. Periods of limited food availability are common in natural environments and have therefore shaped the evolution of highly conserved metabolic and cellular responses aimed at maintaining energy homeostasis and survival [1]. During nutrient deficiency, organisms activate adaptive mechanisms that include mobilization of internal energy reserves, suppression of energetically demanding processes such as growth and reproduction, and induction of stress–response pathways that promote cellular survival [2]. At the cellular level, these responses are coordinated through conserved signaling networks, including pathways which regulate metabolism, autophagy, and stress resistance [3,4]. Because of this evolutionary conservation, starvation responses observed in simple organisms often reflect mechanisms that are also relevant in higher animals.

Understanding starvation physiology has therefore become an important topic not only in ecology and evolutionary biology but also in biomedical research. Nutrient deprivation is increasingly studied in the context of metabolic disorders, developmental programming, aging, and disease-related conditions characterized by energy imbalance, including cachexia and malnutrition [5,6]. Experimental models that allow controlled investigation of starvation-induced responses are essential for unraveling the complex physiological and molecular processes being involved. While vertebrate models have traditionally dominated such studies, there is growing interest in simpler aquatic invertebrate systems that provide experimental accessibility, rapid life cycles, and reduced ethical constraints [7,8]. These models enable the investigation of stress physiology at both organismal and molecular levels while maintaining a high degree of evolutionary relevance.

Among aquatic invertebrates, the brine shrimp *Artemia franciscana* has emerged as a valuable experimental organism in studies of environmental stress, developmental biology, and physiology. This species is widely distributed in hypersaline environments and exhibits remarkable tolerance to extreme conditions such as salinity fluctuations, hypoxia, and environmental stressors [9]. Several biological features make *A. franciscana* a suitable model organism for controlled laboratory studies, including the availability of dormant cysts, synchronous hatching, rapid development, and ease of cultivation. Moreover, both larval and adult stages can be readily observed and quantified using morphometric and behavioral approaches, allowing detailed assessment of physiological responses to environmental perturbations [10,11]. Previous studies have demonstrated that locomotor behavior, growth dynamics, and survival in *Artemia* are sensitive indicators of environmental stress, making these parameters useful phenotypic readouts in experimental studies [12].

Despite the extensive use of *Artemia* in toxicological and ecological research, its potential as a model for studying starvation-induced physiological responses remains insufficiently explored, particularly in the context of stage-associated effects. In many aquatic organisms, starvation can influence development, metabolism, and behavior in a stage-associated manner, reflecting differences in energy requirements and physiological priorities during ontogeny [13,14]. Early developmental stages are often characterized by rapid growth and reliance on endogenous energy reserves, whereas later stages may display more complex behavioral and metabolic responses to nutrient limitation. Understanding how starvation affects different developmental stages within the same organism may therefore provide important insights into adaptive strategies of energy allocation and survival [15].

In addition to organism-level responses, starvation-induced stress is associated with activation of conserved molecular pathways involved in nutrient sensing and cellular adaptation. Comparative analyses across species have demonstrated that signaling networks regulating autophagy, metabolic regulation, and stress resistance are highly conserved throughout evolution [16,17]. Integrating phenotypic observations with pathway-level analysis may therefore help bridge the gap between organismal physiology and underlying molecular mechanisms. Such integrative approaches are increasingly recognized as valuable tools for establishing experimentally tractable model systems that can inform broader biological questions. Due to the limited availability of annotated genomic resources for *Artemia franciscana*, comparative approaches using phylogenetically related crustaceans can provide valuable insights into conserved molecular mechanisms. In this context, *Daphnia pulex* represents a well-characterized model organism with available genomic and protein interaction datasets, enabling the identification of conserved pathways associated with nutrient stress. Therefore, the use of *Daphnia* as a proxy allows hypothesis-driven exploration of molecular processes that may underlie observed phenotypic responses in *Artemia*.

Importantly, differences in starvation responses between developmental stages may be influenced not only by intrinsic ontogenetic factors but also by prior nutritional history. Early-life nutritional conditions are known to affect metabolic capacity, stress resistance, and behavioral performance in later stages. Therefore, distinguishing between purely stage-associated effects and those shaped by developmental nutritional programming represents a critical consideration in the interpretation of experimental results.

The aim of this study was to investigate stage-associated responses to nutrient deprivation in *Artemia franciscana*. Specifically, we examined the effects of acute starvation in newly hatched nauplii as a model of early-life nutrient deprivation, and in adult individuals that had previously been maintained under conditions of minimal caloric intake during development. This experimental design allowed us to assess not only the immediate effects of starvation, but also how prior nutritional history modulates physiological responses to subsequent nutrient deprivation, acknowledging that these factors may interact. In addition, comparative bioinformatic analyses were performed to explore conserved molecular pathways associated with starvation responses.

## 2. Results

### 2.1. Mortality Across Developmental Stages

Mortality rates of *Artemia franciscana* were assessed at 24 h intervals under starvation conditions in both newly hatched nauplii and adult individuals. The results are summarized in Table 1.

In nauplii, no mortality was observed after 24 h of starvation. After 48 h, mortality remained minimal (1%) and did not differ significantly from earlier time points. However, prolonged starvation resulted in a marked increase in mortality, reaching 73% after 72 h (* *p* < 0.001 vs. control).

In contrast, adult individuals exhibited substantially higher sensitivity to starvation. Mortality reached 15% after 24 h (* *p* < 0.05 vs. control), increased to 37.5% after 48 h (*** *p* < 0.001 vs. control), and further rose to 85% after 72 h (*** *p* < 0.001 vs. control). Due to the extremely low survival rate, individuals exposed to 72 h starvation were excluded from subsequent analyses.

It should be noted that, in the adult experiment, control groups represent refeeding following prior developmental nutritional restriction, whereas food-deprived groups represent continued starvation.

Direct comparison between developmental stages revealed significantly higher mortality in adult individuals compared to nauplii at both 24 h (* *p* < 0.05) and 48 h (*** *p* < 0.001). At 72 h, mortality rates were similarly high in both groups, and no statistically significant difference was observed.

### 2.2. Starvation Effects in Newly Hatched Artemia franciscana Nauplii

#### 2.2.1. Morphometric Parameters

Morphometric parameters including total body length and antenna length were measured in control and starved nauplii after 24 h, 48 h and 72 h.

**Figure 1:**
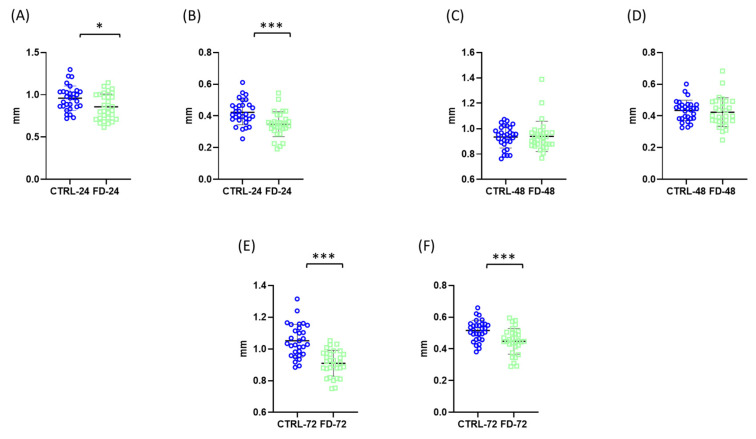
Effects of starvation on morphometric parameters (body length and antennal length) of *Artemia franciscana* nauplii. Morphometric analysis included measurements of total body length and antennal length at different time points: after 24 h (**A**,**B**), 48 h (**C**,**D**), and 72 h (**E**,**F**) of starvation. Within each time point, total body length is shown in panels (**A**,**C**,**E**), while antennal length is shown in panels (**B**,**D**,**F**). Individual data points represent measurements from 30 randomly selected individuals per group, while the total number of organisms per experimental group was 100. Experimental groups are marked as CTRL (fed control) and FD (food-deprived) with respective time point. Data are presented as mean ± SD. Statistical significance between control and starved groups was determined using Student’s *t*-test or Mann–Whitney U test (if non-normal distribution). Differences were considered significant at * *p* < 0.05 and *** *p* < 0.001.

After 24 h of starvation, body length in the control group was 0.9593 ± 0.1455 mm, whereas starved individuals measured 0.8570 ± 0.1522 mm, indicating a significant reduction in growth (Figure 1A). A similar trend was observed for antenna length, which decreased from 0.4225 ± 0.07782 mm in controls to 0.3472 ± 0.07038 mm in starved animals (Figure 1B).

After 48 h, no statistically significant differences were detected between control and starved groups in either body length or antenna length (Figure 1C,D). At 72 h, starvation resulted in a significant reduction in body length (1.051 ± 0.1037 mm in controls vs. 0.9064 ± 0.08434 mm in starved animals), and a significant difference was also observed in antennal length (0.515 ± 0.065 mm in controls vs. 0.441 ± 0.083 mm in starved animals) (Figure 1E,F).

#### 2.2.2. Locomotor Parameters

Locomotor activity was evaluated by measuring distance travelled, average velocity, and maximum velocity.

**Figure 2:**
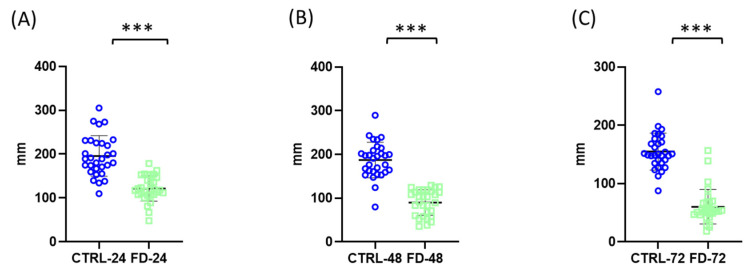
Effects of starvation on locomotor activity (distance travelled) of *Artemia franciscana* nauplii. Total distance travelled was measured after 24 h (**A**), 48 h (**B**), and 72 h (**C**) of starvation. Individual data points represent measurements from 30 randomly selected individuals per group, while the total number of organisms per experimental group was 100. Experimental groups are marked as CTRL (fed control) and FD (food-deprived) with respective time point. Data are presented as mean ± SD. Statistical significance between control and starved groups was determined using Student’s *t*-test or Mann–Whitney U test (if non-normal distribution). Differences were considered significant at *** *p* < 0.001.

After 24 h, the control group travelled 195.7 ± 49.80 mm, whereas starved nauplii travelled significantly shorter distances (122.1 ± 29.20 mm) (Figure 2A).

After 48 h, the reduction in locomotor activity became even more pronounced, with the control group travelling 186.6 ± 41.28 mm compared with 90.06 ± 29.59 mm in the starved group (Figure 2B).

At 72 h, starved animals travelled 60.44 ± 31.21 mm, significantly less than the 155.1 ± 32.19 mm observed in controls (Figure 2C).

**Figure 3:**
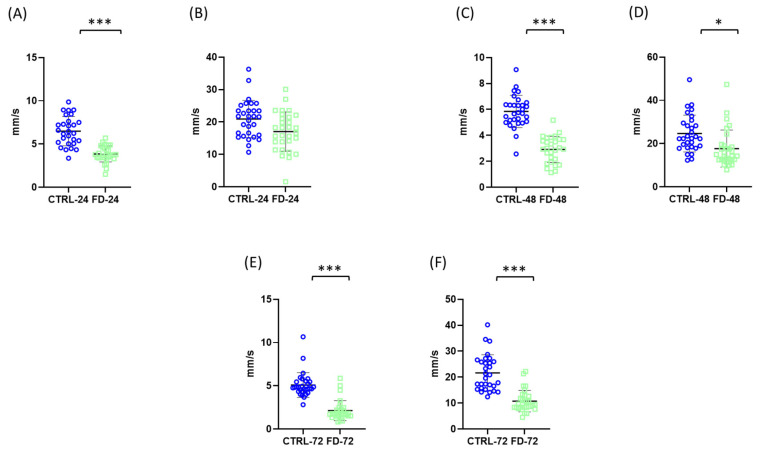
Effects of starvation on locomotor activity (average and maximum swimming velocity) of *Artemia franciscana* nauplii. Average swimming velocity (**A**,**C**,**E**) and maximum swimming velocity (**B**,**D**,**F**) were measured after 24 h (**A**,**B**), 48 h (**C**,**D**), and 72 h (**E**,**F**) of starvation. Individual data points represent measurements from 30 randomly selected individuals per group, while the total number of organisms per experimental group was 100. Experimental groups are marked as CTRL (fed control) and FD (food-deprived) with respective time point. Data are presented as mean ± SD. Statistical significance between control and starved groups was determined using Student’s *t*-test or Mann–Whitney U test (if non-normal distribution). Differences were considered significant at * *p* < 0.05 and *** *p* < 0.001.

Analysis of locomotor performance revealed significant differences between control and starved groups in both average and maximum swimming velocity. After 24 h, the average velocity in the control group was 6.455 ± 1.656 mm/s, whereas starved nauplii exhibited a significantly lower value of 3.872 ± 0.9458 mm/s (Figure 3A). In contrast, no statistically significant difference was observed in maximum velocity at this time point, (Figure 3B).

After 48 h of starvation, both locomotor parameters were significantly reduced in the starved group. The average velocity decreased from 5.827 ± 1.262 mm/s in the control group to 2.919 ± 1.028 mm/s in the starved group, while the maximum velocity declined from 24.64 ± 8.786 mm/s to 18.33 ± 9.009 mm/s (Figure 3C,D).

A similar pattern was observed after 72 h of starvation. The average velocity in the control group was 5.100 ± 1.455 mm/s, compared with 2.145 ± 1.219 mm/s in the starved group. Maximum velocity also showed a pronounced reduction, decreasing from 21.97 ± 7.115 mm/s in controls to 10.95 ± 4.242 mm/s in starved individuals (Figure 3E,F).

### 2.3. Effects of Starvation in 8 Dph Artemia franciscana with Prior Nutrient Defficiency

#### 2.3.1. Morphometric Parameters

Morphometric parameters were analyzed in order to evaluate the effects of starvation on growth dynamics in adult *Artemia franciscana*. Body length and antenna length were measured after 24 h and 48 h of complete nutrient restriction. Due to the extremely low survival rate, individuals exposed to 72 h starvation were excluded from further analyses.

**Figure 4:**
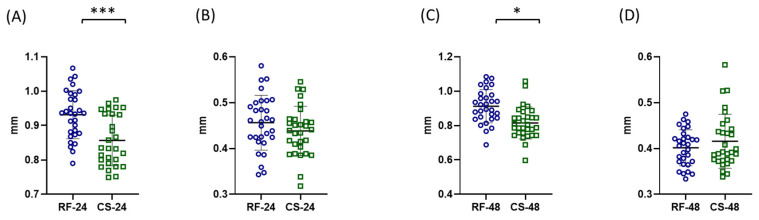
Effects of starvation on morphometric parameters (body length and antennal length) of adult *Artemia franciscana*. Morphometric analysis included measurements of total body length and antennal length after 24 h (**A**,**B**) and 48 h (**C**,**D**) of starvation. Total body length is presented in panels (**A**,**C**), while antennal length is presented in panels (**B**,**D**). Individual data points represent measurements from 30 randomly selected individuals per group, while the total number of organisms per experimental group was 100. Experimental groups are marked as RF (refed control) and CS (continued starvation) with respective time point. Data are presented as mean ± SD. Statistical significance between control and starved groups was determined using Student’s *t*-test or Mann–Whitney U test (if non-normal distribution). Differences were considered significant at * *p* < 0.05 and *** *p* < 0.001.

After 24 h of starvation, the mean body length of refed individuals was 0.931 ± 0.07 mm, whereas starved individuals showed a significantly lower value of 0.856 ± 0.07 mm (Figure 4A).

A similar pattern was observed after 48 h of starvation, where the mean body length of the refed group was 0.912 ± 0.09 mm, compared with 0.82 ± 0.1 mm in the starved group (Figure 4C).

In contrast, antenna length did not show statistically significant differences between refed control and starved groups at either time point. After 24 h, the mean antenna length was 0.456 ± 0.006 mm in the refed control group and 0.4382 ± 0.054 mm in the starved group (Figure 4B). Similarly, after 48 h the antenna lengths were 0.402 ± 0.039 mm and 0.42 ± 0.059 mm in refed control and starved individuals, respectively (Figure 4D).

#### 2.3.2. Locomotor Parameters

**Figure 5:**
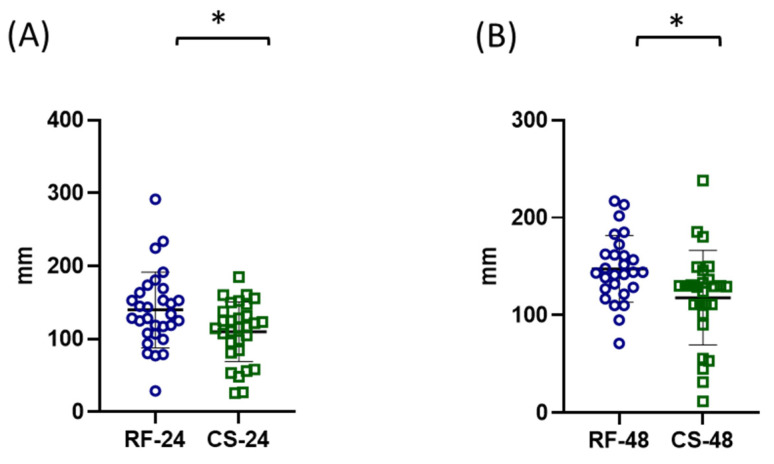
Effects of starvation on locomotor activity (distance travelled) of adult *Artemia franciscana*. Total distance travelled was measured after 24 h (**A**) and 48 h (**B**) of starvation. Individual data points represent measurements from 30 randomly selected individuals per group, while the total number of organisms per experimental group was 100. Experimental groups are marked as RF (refed control) and CS (continued starvation) with respective time point. Data are presented as mean ± SD. Statistical significance between control and starved groups was determined using Student’s *t*-test or Mann–Whitney U test (if non-normal distribution). Differences were considered significant at * *p* < 0.05.

The analysis of distance travelled showed a significant reduction in starved individuals. After 24 h, the refed control group travelled 139.7 ± 51.9 mm, whereas the starved group travelled 110 ± 40.86 mm (Figure 5A). After 48 h, the distance travelled was 147.5 ± 34.19 mm in the refed control group and 117.8 ± 50.24 mm in the starved group (Figure 5B).

**Figure 6:**
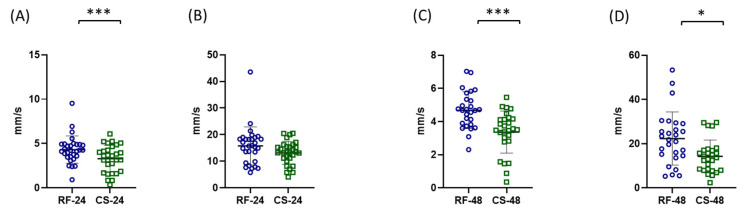
Effects of starvation on locomotor activity (average and maximum swimming velocity) of adult *Artemia franciscana*. Average swimming velocity (**A**,**C**) and maximum swimming velocity (**B**,**D**) were measured after 24 h (**A**,**B**) and 48 h (**C**,**D**) of starvation. Individual data points represent measurements from 30 randomly selected individuals per group, while the total number of organisms per experimental group was 100. Experimental groups are marked as RF (refed control) and CS (continued starvation) with respective time point. Data are presented as mean ± SD. Statistical significance between control and starved groups was determined using Student’s *t*-test or Mann–Whitney U test, (if non-normal distribution). Differences were considered significant at * *p* < 0.05 and *** *p* < 0.001.

A similar trend was observed for average swimming velocity. After 24 h, the refed control group exhibited an average velocity of 4.31 ± 1.5 mm/s, compared with 3.29 ± 1.4 mm/s in the starved group (Figure 6A). After 48 h of starvation, the refed control group reached 4.68 ± 1.1 mm/s, whereas the starved group showed a significantly lower value of 3.343 ± 1.31 mm/s (Figure 6C).

The analysis of maximum swimming velocity revealed that after 24 h of starvation the refed control group reached 15.57 ± 7.36 mm/s, while the starved group reached 13.35 ± 4.5 mm/s, with no statistically significant difference between groups (Figure 6B). However, after 48 h of starvation, maximum velocity was significantly reduced in starved individuals. The refed control group exhibited a maximum velocity of 22.34 ± 12.04 mm/s, compared with 14.43 ± 7.65 mm/s in the starved group (Figure 6D).

#### 2.3.3. Recovery After Restoration of Feeding

Because adult experimental individuals had been maintained under conditions of minimal caloric intake during development, an additional recovery analysis was performed after restoration of feeding to assess whether the observed starvation-related changes were reversible and whether renewed nutrient availability could partially restore locomotor and morphometric performance.

**Figure 7:**
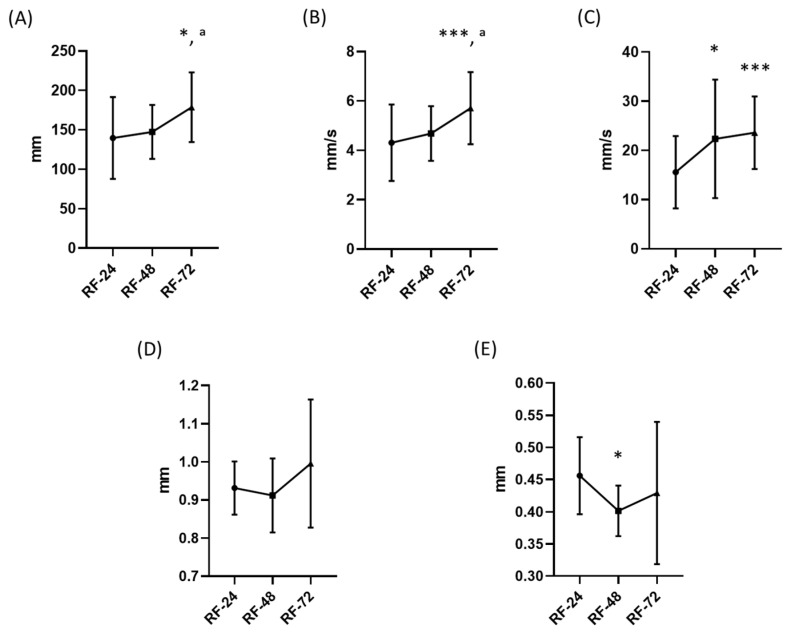
Effects of feeding restoration on locomotor and morphometric parameters of adult *Artemia franciscana*. Locomotor activity was assessed as total distance travelled (**A**), average swimming velocity (**B**), and maximum swimming velocity (**C**) following restoration of feeding after a period of minimal caloric intake. Morphometric parameters included total body length (**D**) and antennal length (**E**). Measurements were performed at defined time points after refeeding (24 h, 48 h, and 72 h). Individual data points represent measurements from 30 randomly selected individuals per group, while the total number of organisms per experimental group was 100. Experimental groups are marked as RF (refed control) and CS (continued starvation) with respective time point represented with different symbols (cirlces, squares and triangles). Data are presented as mean ± SD. Statistical significance was determined using one-way ANOVA followed by Tukey’s post hoc test or Kruskal–Wallis test with Dunn’s post hoc test (for non-parametric data). Differences were considered significant at * *p* < 0.05, and *** *p* < 0.001. Asterisks (*) indicate statistically significant differences compared to the 24 h control group, while superscript letters (^a^) indicate statistically significant differences compared to the 48 h control group.

Analysis of locomotor parameters showed a partial recovery response following restoration of feeding. Average and maximum swimming velocity increased significantly after refeeding, whereas total distance travelled showed a significant increase only after 72 h (Figure 7A–C).

Morphometric analysis indicated that total body length remained unchanged throughout the examined recovery period. In contrast, antennal length was significantly reduced after 48 h, but returned to values comparable to the control group after 72 h of feeding restoration (Figure 7D,E).

Given the relatively high variability observed in several parameters, these findings should be interpreted as indicative of partial functional recovery rather than complete compensatory restoration. The observed variability likely reflects biological heterogeneity in the response to nutrient reintroduction, as well as differences in individual physiological status following developmental nutritional restriction. Since all individuals were subjected to developmental nutritional restriction prior to this phase, the observed differences reflect relative responses to nutrient reintroduction rather than comparisons with fully nourished baseline conditions.

### 2.4. In Silico Analysis of Starvation-Related Pathways

#### 2.4.1. Identification of Autophagy-Related Genes in *Daphnia pulex*

To investigate molecular mechanisms potentially involved in starvation responses, an in silico identification of autophagy-related genes was performed in *Daphnia pulex* using a homology-based approach. Protein sequences of well-characterized autophagy components from model organisms were retrieved and used as queries for BLAST (v2.17.0, https://blast.ncbi.nlm.nih.gov/Blast.cgi, accessed on 10 January 2026) searches against the *D. pulex* proteome. Homologous sequences corresponding to core components of the canonical autophagy pathway were identified based on sequence similarity and conserved functional domains. The identified candidates included genes associated with the principal stages of autophagy, including: autophagy initiation, represented by components of the *ATG1*/*ULK* kinase complex; vesicle nucleation, involving members of the *VPS34* phosphatidylinositol 3-kinase complex; phagophore membrane elongation, mediated by the *ATG5–ATG12–ATG16* conjugation system; *LC3/ATG8* lipidation machinery, responsible for autophagosome membrane expansion and maturation. In total, 25 candidate autophagy-related genes were identified, representing the major functional modules of the conserved autophagy machinery (Appendix A—Table A1).

#### 2.4.2. Protein–Protein Interaction Network Analysis

To assess potential functional relationships among the identified genes, a protein–protein interaction (PPI) network was constructed using the STRING database.

The resulting network comprised 24 nodes and 164 edges, with an average node degree of 13.7, indicating a highly interconnected interaction structure.

**Figure 8:**
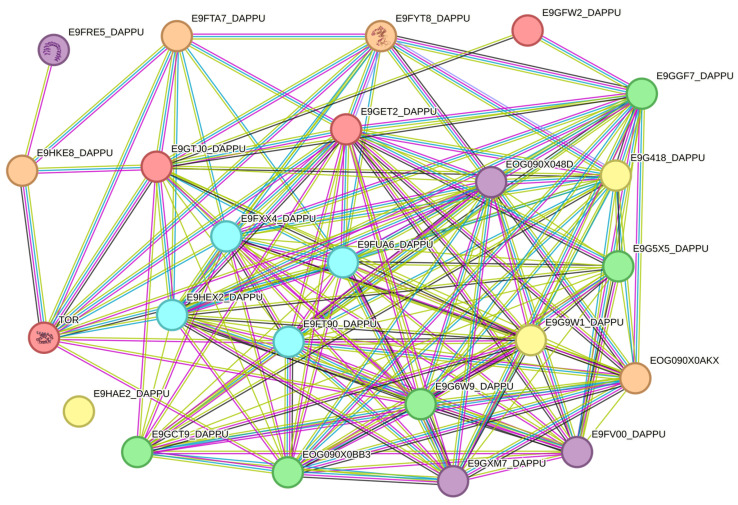
Protein–protein interaction (PPI) network of selected genes associated with the analyzed biological processes. Nodes are colored according to their functional roles within the autophagy pathway: red—nutrient sensing and TOR signaling, orange—autophagy initiation, blue—vesicle nucleation, green—phagophore elongation, purple—*LC3/ATG8* lipidation machinery, yellow—autophagosome maturation and trafficking.

Importantly, the number of observed interactions was significantly higher than expected for a random set of proteins of similar size (expected number of edges = 3; PPI enrichment *p* < 1.0 × 10^−16^), indicating strong functional connectivity within the gene set. Topological inspection of the network suggested a dense interaction cluster involving components of the TOR signaling pathway and the core autophagy machinery, suggesting coordinated regulation of nutrient sensing and autophagic processes (Figure 8).

#### 2.4.3. Enrichment Analysis of Identified Genes

To further characterize the biological relevance of the identified genes, Gene Ontology (GO) enrichment analysis was performed.

**Figure 9:**
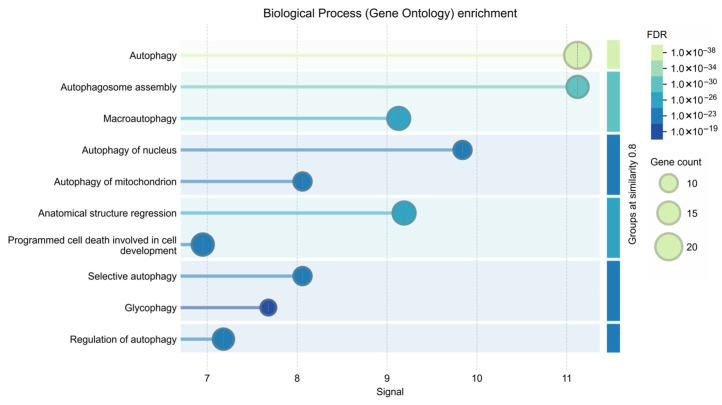
Gene Ontology (GO) enrichment analysis of the analyzed gene set—Biological Process category. Bubble plot representing significantly enriched biological processes associated with the analyzed genes. The *x*-axis represents the enrichment ratio, while the *y*-axis shows the enriched GO terms. Bubble size corresponds to the number of genes involved in each term (gene count), and color intensity represents the adjusted *p*-value expressed as the false discovery rate (FDR).

Significant enrichment was observed for biological processes associated with autophagy and cellular adaptation to nutrient deprivation. The most strongly enriched GO biological process terms included: macroautophagy, regulation of autophagy, autophagosome organization, cellular response to starvation (Figure 9).

**Figure 10:**
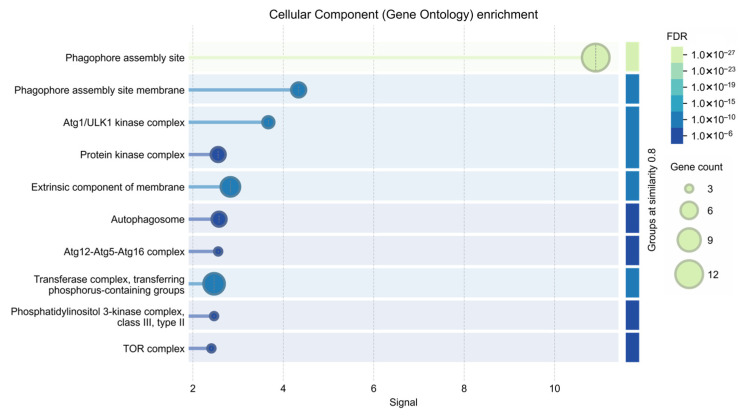
Gene Ontology (GO) enrichment analysis of the analyzed gene set—Molecular Function category. Bubble plot illustrating significantly enriched molecular functions associated with the analyzed genes. The *x*-axis represents the enrichment ratio, while the *y*-axis shows the corresponding GO terms. Bubble size reflects the number of genes associated with each function, while color intensity corresponds to the false discovery rate (FDR).

Enrichment analysis of cellular component terms suggested strong association with intracellular structures involved in autophagic activity, including: autophagosome, pre-autophagosomal structure, *TOR* protein complex (Figure 10).

**Figure 11:**
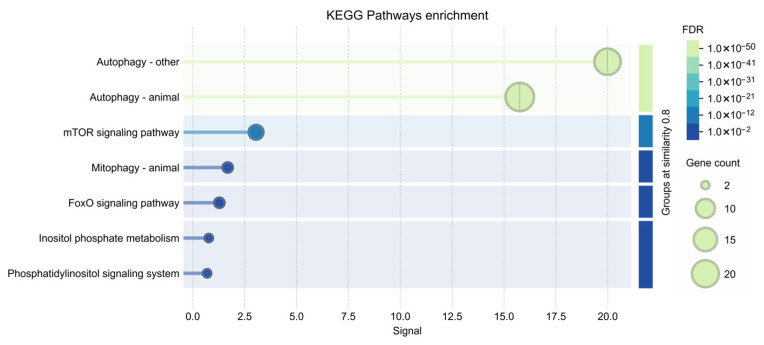
KEGG pathway enrichment analysis of the analyzed gene set. Bubble plot showing significantly enriched KEGG pathways associated with the analyzed genes. The *x*-axis represents the enrichment ratio, while the *y*-axis lists the enriched pathways. Bubble size indicates the number of genes involved in each pathway, and the color gradient represents the statistical significance based on the false discovery rate (FDR).

Additionally, KEGG pathway analysis suggested significant enrichment of pathways related to autophagy and TOR signaling, further supporting the involvement of the identified gene set in nutrient-sensing and autophagy-associated regulatory mechanisms (Figure 11).

## 3. Discussion

Starvation represents a fundamental ecological stressor that affects growth, metabolism, and behavioral activity in aquatic invertebrates. Organisms exposed to nutrient deprivation activate physiological adaptations aimed at conserving energy and prolonging survival under conditions of limited resource availability [18]. Understanding how these mechanisms manifest in simple experimental models may provide insight into conserved strategies of metabolic adaptation across different taxa.

A pronounced stage-associated difference in starvation sensitivity was observed in our experiments, particularly in relation to mortality rates. While newly hatched nauplii exhibited minimal mortality during the first 48 h of starvation, adult individuals displayed a much more rapid increase in lethality. Early developmental stages of *Artemia* rely heavily on endogenous yolk platelet reserves, which serve as a primary metabolic energy source during the first days after hatching and allow larvae to maintain physiological processes even in the absence of external nutrients [19,20]. Similar starvation resistance in early larval stages has been reported in several aquatic invertebrates, where endogenous energy stores buffer short-term nutritional stress [21]. The lower mortality observed in nauplii compared with adults therefore probably reflects the availability of yolk-derived energy reserves that temporarily sustain metabolic activity during early starvation. Although the observed differences between groups are consistent with stage-related variation in starvation responses, these findings should also be interpreted in the context of prior nutritional history, as adult individuals in this study were subjected to developmental nutritional restriction.

In adult individuals, starvation induced pronounced effects on survival. However, unlike newly hatched nauplii, adult organisms in this study had previously experienced prolonged minimal caloric intake during development. Consequently, the observed responses likely represent the cumulative effects of chronic developmental nutritional limitation and subsequent acute starvation. Previous studies have demonstrated that early-life nutritional stress can have long-term consequences on growth, metabolic efficiency, and behavioral performance in later life stages [22,23]. Such developmental programming may increase susceptibility to additional stressors, including nutrient deprivation. The markedly higher mortality observed in adult individuals compared to nauplii further supports this interpretation. While nauplii benefit from endogenous yolk reserves that buffer short-term starvation, adults lack such internal energy sources and rely entirely on external nutrient availability. When combined with prior developmental caloric restriction, this may result in reduced metabolic resilience and accelerated depletion of energy reserves, ultimately leading to increased mortality.

Morphometric analysis revealed that starvation significantly affected body growth in both developmental stages, although the magnitude and temporal pattern differed. In newly hatched nauplii, significant reductions in body length were observed after 24 h and again after prolonged starvation at 72 h, whereas no statistically significant difference was detected at the intermediate 48 h time point. Growth suppression during nutrient deprivation has been widely documented across aquatic organisms and reflects a metabolic shift in which anabolic processes such as tissue growth are downregulated in order to conserve energy for essential cellular functions [24,25,26]. Similar reductions in body size during starvation have been reported in fish and crustacean species exposed to nutrient limitation [27,28]. The transient absence of morphometric differences at 48 h in nauplii may indicate that endogenous yolk reserves temporarily sustain developmental growth during this intermediate stage before becoming depleted during prolonged starvation. In adult individuals, starvation produced a consistent reduction in body length already after 24 h and 48 h of nutrient deprivation. Unlike larvae, adult organisms must allocate energy primarily toward maintaining physiological stability rather than supporting developmental growth processes. Previous studies have demonstrated that adult animals exposed to starvation often exhibit rapid reductions in body mass and structural growth due to activation of catabolic pathways that mobilize stored nutrients especially if they were exposed to prior nutrient deficiency [22]. The reduced body length observed in adult *Artemia franciscana* therefore may reflects accelerated mobilization of metabolic reserves required to maintain survival under starvation conditions. Interestingly, antenna length did not exhibit significant changes under starvation conditions in either developmental stage. Morphological structures that play essential roles in environmental sensing and locomotion are often preserved during metabolic stress because they remain critical for behavioral responses such as food searching or predator avoidance. Previous work has suggested that during nutrient limitation organisms may selectively prioritize maintenance of functional structures necessary for survival while suppressing overall somatic growth [29,30,31]. This may explain why antenna morphology remained relatively stable despite the observed reductions in body length.

Starvation also produced strong effects on locomotor behavior, which appeared to be among the most sensitive physiological indicators of nutrient stress. Both nauplii and adult individuals showed significant reductions in distance travelled and swimming velocity under starvation conditions. Behavioral suppression during starvation has been described in numerous animal models and is widely interpreted as an energy conservation strategy aimed at reducing metabolic expenditure [32,33,34]. Experimental studies have demonstrated that animals often decrease locomotor activity in response to nutrient deprivation in order to prolong survival and delay exhaustion of energy reserves [35,36]. The reductions in locomotor activity observed in our experiments therefore likely represent adaptive behavioral responses that limit energy consumption during starvation. Locomotor responses were particularly evident in newly hatched nauplii, where reductions in swimming activity occurred already after 24 h of starvation despite relatively low mortality rates. Behavioral changes are often among the earliest responses to metabolic stress and may precede structural or survival-related effects. Previous studies have shown that starvation can rapidly alter movement patterns and activity levels in animals exposed to nutrient limitation [18]. This suggests that locomotor activity may serve as a sensitive early indicator of metabolic stress in *Artemia franciscana*. An additional aspect of our experimental design involved examining adult individuals that had previously experienced minimal caloric intake during development. Behavioral suppression under starvation conditions is a well-established energy-saving strategy [35,37], but its magnitude may be amplified in organisms with compromised energetic status due to prior nutritional limitation. Together, these findings suggest that adult starvation responses in *A.franciscana* are strongly influenced by nutritional history, highlighting the importance of considering developmental context when interpreting physiological responses to environmental stress. Nutritional conditions during early life are known to influence adult physiological performance and stress tolerance [23]. Studies in insects and aquatic organisms have shown that early nutritional stress can produce long-term effects on growth rate, locomotor performance, and metabolic regulation in later life stages [37,38].

Interestingly, the restoration of feeding produced signs of partial physiological recovery. In control groups, increases in locomotor parameters and antenna length were observed following renewed nutrient availability. Compensatory growth and recovery following starvation are well-documented phenomena across aquatic organisms and represent adaptive responses allowing individuals to restore physiological performance after periods of resource scarcity [39,40]. Such compensatory mechanisms may enable organisms to recover from temporary nutritional stress and improve survival in environments characterized by fluctuating food availability.

An important limitation of the present study concerns the interpretation of starvation effects in adult individuals. Future studies incorporating fully fed developmental conditions prior to adult starvation will be necessary to disentangle the independent and interactive contributions of developmental and acute nutritional stress. The observed changes following refeeding should be interpreted as relative functional improvement rather than complete recovery to baseline conditions, particularly given that all individuals were previously subjected to developmental nutritional restriction.

Beyond organism-level phenotypes, the bioinformatic analysis performed in this study provided insight into the molecular pathways potentially underlying starvation responses. It is important to emphasize that the bioinformatic analysis performed in this study is based on a proxy species and should be interpreted as hypothesis-generating rather than direct functional validation. Homology-based identification of autophagy-related genes suggested a conserved set of proteins associated with key functional modules of the canonical autophagy machinery. Autophagy represents a fundamental cellular process activated during nutrient deprivation and functions to recycle intracellular components in order to sustain cellular metabolism under conditions of energy limitation [3,4]. The identification of 25 candidate autophagy-related genes in the crustacean model suggests that starvation responses observed at the organismal level may be supported by highly conserved cellular stress–response pathways.

Protein–protein interaction network analysis further indicated strong functional connectivity among the identified genes. The observed interaction network exhibited significantly more connections than expected by chance, indicating that the identified proteins likely participate in coordinated regulatory processes. Importantly, central nodes within the network were associated with TOR signaling and core autophagy machinery, both of which play essential roles in nutrient sensing and metabolic adaptation. The mTOR pathway acts as a central regulator of cellular metabolism by integrating signals related to nutrient availability and energy status, while autophagy functions as a downstream adaptive mechanism activated during nutrient scarcity [3].

Functional enrichment analysis indicates significant overrepresentation of biological processes related to autophagy, autophagosome organization, and cellular response to starvation. These findings are consistent with previous studies indicating that nutrient deprivation triggers activation of autophagy-related pathways across diverse taxa [41]. Taken together, the bioinformatic results support the idea that the phenotypic responses observed in *Artemia franciscana* including altered locomotion, growth suppression, and increased mortality, may be linked to conserved molecular pathways involved in nutrient sensing and cellular stress adaptation.

It is important to note that the bioinformatic analysis was not performed directly on *Artemia franciscana* but on a phylogenetically related crustacean species. While these findings are consistent with conserved roles of autophagy and *TOR* signaling in nutrient stress responses, experimental validation in *Artemia* will be required to confirm their functional involvement.

Although this study was conducted in a non-mammalian model organism, the observed responses to prolonged nutrient deprivation and subsequent refeeding may have broader biological and clinical relevance. In particular, the differential sensitivity of developmental stages to starvation, as well as the partial recovery observed following restoration of feeding, are consistent with the concept of metabolic programming and adaptive responses to nutritional stress described in higher organisms. In clinical and human health contexts, prolonged caloric restriction or intermittent fasting is often explored for its potential metabolic benefits. However, our findings suggest that extended or poorly controlled nutrient deprivation may lead to pronounced functional impairments and incomplete recovery, particularly when occurring during critical developmental periods. These results align with evidence indicating that early-life nutritional stress can have long-lasting effects on metabolism, growth, and stress resilience. Therefore, while short-term dietary interventions may have beneficial effects under controlled conditions, prolonged or severe nutritional restriction may carry potential risks, especially in vulnerable developmental stages. Our results emphasize the importance of balanced nutritional strategies and highlight the need for further research into the long-term consequences of starvation and refeeding across different biological systems.

## 4. Materials and Methods

### 4.1. Experimental Organisms and Culture Conditions

Cysts of *A. franciscana* (Sera GmbH, Heinsberg, Germany) were stored at 4 °C in darkness until use. For hatching, 500 mg of cysts were incubated in 1 L of artificial seawater under continuous aeration and illumination (1200 lux). Nauplii emerged within 18–24 h post-incubation. During the first 24 h post-hatching, organisms relied on endogenous yolk reserves. After yolk depletion, individuals were transferred to enriched medium and maintained at a density of 98 ± 10 individuals/mL to minimize crowding stress and ensure standardized growth conditions [42,43].

### 4.2. Experimental Design and Starvation Protocol

To assess starvation responses during early development, newly hatched *Artemia franciscana* nauplii were assigned to six experimental groups, each containing 100 individuals (n = 100 per group). The groups were defined according to feeding condition and exposure duration as follows: CTRL-24, CTRL-48, CTRL-72, FD-24, FD-48, and FD-72. Each group was distributed into four culture vessels containing 50 mL of artificial seawater (Reef Salt, Aquaforest, Brzesko, Poland), with 25 organisms per vessel, in order to reduce density-dependent effects and maintain stable culture conditions [43,44]. Culture vessels were used as technical replicates to ensure stable environmental conditions and reduce density-dependent variability. For all analyses, individual organisms were treated as biological replicates, as measurements were performed at the level of single individuals. Control groups (CTRL-24, CTRL-48, CTRL-72) were maintained under standard feeding conditions, consisting of yeast supplementation (Centroprom, Belgrade, Serbia) at a concentration of 50 mg L^−1^, while food-deprived groups (FD-24, FD-48, FD-72) were maintained under identical environmental conditions but received no exogenous nutrient supply during the corresponding exposure period. Thus, food deprivation in this experiment refers to complete absence of feeding after hatching. The three control groups and three food-deprived groups were analyzed after 24 h, 48 h, and 72 h, respectively. Each experimental group consisted of 100 individuals (n = 100), which were used for mortality assessment. From each group, 30 individuals were randomly selected for detailed morphometric and locomotor analyses. This phase of the experiment was designed to evaluate the acute effects of starvation during the earliest developmental stage, when nauplii transition from endogenous yolk reserves to external nutrient supply and exhibit high metabolic demands associated with early growth and development. The selected exposure durations of 24 h, 48 h, and 72 h were chosen to represent early, intermediate, and prolonged starvation stress [45,46].

To investigate starvation responses in adult *Artemia franciscana*, an independent cohort of organisms was reared from hatching under conditions of reduced nutritional availability. During the 8-day developmental period, organisms were maintained under a minimal feeding regime consisting of yeast supplementation at a concentration of 5 mg L^−1^ every 48 h. This feeding protocol was designed to support survival while preventing ad libitum growth, thereby simulating chronic nutritional limitation [47,48]. Upon reaching the adult stage (8 days post-hatching), a total of 600 individuals were randomly selected and assigned to six experimental groups (n = 100 per group): RF-24, RF-48, RF-72, CS-24, CS-48, and CS-72. Each group was distributed into four culture vessels (25 individuals per vessel) to minimize density-related effects. Following group allocation, RF groups received standard feeding conditions consisting of yeast supplementation at 50 mg L^−1^, whereas CS groups were subjected to complete food deprivation (no exogenous nutrient supply) for 24 h, 48 h, or 72 h. Thus, the adult experiment compares refeeding (RF groups) versus continued starvation (CS groups) after a period of developmental nutritional restriction. Each adult experimental group consisted of 100 individuals (n = 100) for mortality assessment, of which 30 randomly selected individuals were used for morphometric and locomotor analyses.

To further evaluate the functional consequences of nutrient availability following developmental restriction, an additional analysis was performed focusing on the response to nutrient reintroduction. In addition to comparisons between refed (RF) and continued starvation (CS) groups, temporal changes were also analyzed within the RF groups (24 h, 48 h, and 72 h) to assess whether prolonged refeeding leads to progressive functional improvement. This approach enabled assessment of whether renewed nutrient availability induces measurable changes in locomotor and morphometric parameters over time, relative to sustained deprivation under otherwise identical conditions. Measurements were conducted at defined time points (24 h, 48 h, and 72 h) to capture the temporal dynamics of this response. Although a continuously fed control group was not included in this phase of the study, the RF groups serve as an internal reference for nutrient restoration following developmental restriction, allowing relative evaluation of functional improvement over time.

In this study, adult individuals were intentionally reared under reduced nutritional conditions prior to the starvation experiment in order to evaluate whether developmental nutritional history influences later starvation responses. Nutritional conditions experienced during early development are known to shape metabolic strategies and stress tolerance in many organisms [22,23]. Therefore, this approach allowed us to investigate not only acute starvation effects but also the potential role of developmental nutritional programming in shaping starvation sensitivity.

### 4.3. Mortality Assessment

Mortality was assessed immediately after each starvation exposure period. Individuals were classified as non-viable when they exhibited a complete absence of spontaneous movement and showed no response to external mechanical stimulation. Mechanical stimulation was applied by gently touching the organism with the tip of a pipette to assess potential movement responses. Organisms that did not respond to this stimulus were considered non-viable and recorded as dead. Mortality rates were calculated for each experimental group as the proportion of non-viable individuals relative to the total number of organisms at the beginning of the exposure period [49,50].

### 4.4. Morphometric Measurements

Morphometric analysis was performed using microscopic video recordings of surviving organisms. Thirty individuals were analyzed per group. Recordings were obtained using an inverted microscope (Zeiss Primovert, Oberkochen, Germany) under standardized conditions to ensure consistency across all measurements. All recordings were performed with same magnification on a white background containing a millimeter reference scale, allowing accurate calibration of measurement distances. Images were analyzed using ImageJ software (version 1.54 r; National Institutes of Health, Bethesda, MD, USA). Calibration of the measurement system was performed using the “Set Scale” function within the software based on the reference scale present in the recordings. Morphometric parameters included sagittal body length and the length of the anterior antennae. Measurements were performed by selecting frames in which organisms appeared fully extended, ensuring consistent anatomical positioning during measurement. The measured values were expressed in millimeters and used for quantitative comparisons between experimental and control groups [51].

### 4.5. Locomotor Parameter Analysis

For locomotor activity analysis, thirty individuals were randomly selected from each experimental group across all culture vessels. Each organism was individually recorded for 30 s, and locomotor parameters including distance travelled, average velocity, and maximum velocity were subsequently quantified from the recorded videos. Locomotor activity was assessed from video recordings using the ZembryoAnalyzer software (available at: https://github.com/darkopuflovic/ZembryoAnalyser; accessed on 10 January 2026) platform, which enables automated tracking of organism movement [52]. Recorded videos were imported into the software, which automatically detected moving organisms within the field of view and reconstructed their movement trajectories frame by frame. Background noise and non-moving elements were filtered using the software’s built-in image processing functions, allowing precise identification of the region of interest corresponding to the moving organism. Based on reconstructed trajectories, the software calculated locomotor parameters describing swimming behavior. These parameters included total distance traveled during the recording period as well as average, minimum, and maximum swimming speed. The obtained locomotor metrics provided quantitative estimates of organismal activity under starvation and control conditions.

### 4.6. Comparative Bioinformatic Analysis of Starvation-Related Pathways

#### 4.6.1. Identification of Candidate Autophagy-Related Genes

Genomic and functional annotation resources for *Artemia franciscana* remain limited, a comparative bioinformatic approach was applied using the phylogenetically related crustacean species *Daphnia pulex* as a reference model. Specifically, genes involved in autophagy and nutrient-sensing pathways were first identified in *D. pulex*, and subsequently used to identify the presence of evolutionarily conserved orthologs and pathways potentially relevant to starvation responses in *A. franciscana*.

To explore conserved molecular mechanisms potentially associated with starvation responses, a comparative bioinformatic analysis of autophagy-related pathways was performed. Candidate genes involved in nutrient sensing, metabolic stress responses, and autophagy were identified based on the Kyoto Encyclopedia of Genes and Genomes (KEGG) pathway (https://www.kegg.jp/; accessed on 15 January 2026) related to autophagy and nutrient signaling (KEGG pathway: Autophagy—animal, pathway ID: dpx04136) [53,54].

Reference protein sequences representing key components of the canonical autophagy machinery were retrieved from the NCBI Protein database [55]. These sequences included proteins associated with major functional modules of the autophagy pathway, such as nutrient sensing via *TOR* signaling, autophagy initiation (*ULK/ATG1* complex), vesicle nucleation (*VPS34–Beclin* complex), phagophore elongation (*ATG5–ATG12–ATG16* conjugation system), and *LC3/ATG8* lipidation machinery. These reference proteins were used as query sequences for homology-based identification of evolutionarily conserved orthologs in crustacean species [56,57,58].

#### 4.6.2. Homology Search and Identification of Crustacean Orthologs

Due to the limited availability of annotated genomic resources for *Artemia franciscana*, a homology-based approach was employed to identify candidate genes potentially involved in starvation-related processes. Orthologous proteins were identified using BLASTP (https://blast.ncbi.nlm.nih.gov/Blast.cgi; accessed on 15 January 2026) searches implemented through the National Center for Biotechnology Information (NCBI) BLAST platform [59].

Sequence similarity searches were conducted against publicly available crustacean protein databases in order to detect homologous proteins conserved between model organisms and crustacean species [60].

Candidate orthologs were selected based on the following criteria: E-value ≤ 1 × 10^−5^; minimum sequence identity ≥ 30%; and query coverage ≥ 50% [61].

These thresholds were chosen to ensure reliable identification of homologous proteins while minimizing false-positive matches [62].

Using this approach, a set of 25 candidate genes associated with the core autophagy machinery was identified.

#### 4.6.3. Protein–Protein Interaction Network Reconstruction

To investigate potential functional relationships among the identified proteins, a protein–protein interaction (PPI) network was reconstructed using the STRING database (version 12.0; https://string-db.org/). Because comprehensive interaction datasets are not currently available for *Artemia franciscana*, the crustacean species *Daphnia pulex* was used as a reference organism due to the availability of annotated protein interaction data and its phylogenetic proximity [63,64].

Network topology parameters including: number of nodes, number of edges, average node degree, and PPI enrichment *p*-value were calculated automatically within the STRING platform in order to evaluate whether the observed network connectivity was significantly greater than expected by chance [65,66].

#### 4.6.4. Functional Enrichment Analysis

Functional enrichment analyses were conducted using Gene Ontology (GO) annotations and KEGG pathway analysis available within the STRING platform. These analyses were performed to identify significantly enriched biological processes, cellular components, and molecular pathways associated with the identified starvation-related gene set [63,67,68].

### 4.7. Statistical Analysis

All experimental data were expressed as mean ± standard deviation (SD). Statistical analyses were performed using GraphPad Prism 8 (version 8; GraphPad Software Inc., La Jolla, CA, USA). The normality of data distribution was evaluated using appropriate normality tests prior to further statistical analysis. Comparisons between two independent groups were performed using Student’s *t*-test when data followed a normal distribution, while the Mann–Whitney U test was used for non-normally distributed data. In comparisons with multiple experimental groups, one-way analysis of variance (ANOVA) followed by Tukey’s post hoc test was used for normally distributed datasets. In cases where data deviated from normal distribution, the Kruskal–Wallis test followed by Dunn’s post hoc test was applied. Mortality data were analyzed as endpoint proportions using Fisher’s exact test, with the number of dead individuals expressed as a percentage of the total number of organisms per group. Statistical significance was considered at *p* < 0.05, while highly significant differences were defined as *p* < 0.001.

### 4.8. Ethical Considerations

The present study involved experiments on aquatic invertebrates (*Artemia franciscana*), which are not subject to institutional ethical approval requirements in many jurisdictions. All experimental procedures were conducted in accordance with standard laboratory practices for the maintenance and handling of aquatic invertebrate organisms.

### 4.9. Use of Artificial Intelligence Tools

Generative artificial intelligence tools were not used for data generation, analysis, or interpretation in this study. Artificial intelligence tools were used solely for minor linguistic editing of the manuscript text.

## 5. Conclusions

Starvation induces distinct physiological responses in *Artemia franciscana*, affecting survival, growth dynamics, and locomotor activity. Newly hatched nauplii exhibited relatively high tolerance to short-term nutrient deprivation, likely due to endogenous energy reserves, whereas adult individuals showed increased sensitivity, reflected in higher mortality and reduced locomotor performance. Importantly, these differences should be interpreted in the context of both developmental stage and prior nutritional history, as adult individuals in this study were subjected to prolonged nutritional limitation during development. Following nutrient reintroduction, partial improvements in locomotor activity and selected morphometric parameters were observed, indicating a degree of functional recovery, however, these effects remained incomplete and variable, reflecting differences in individual physiological status.

Bioinformatic analysis suggested the presence of conserved autophagy-related pathways associated with nutrient stress responses. However, these findings are based on a proxy species and should be considered hypothesis-generating rather than direct functional evidence.

A key limitation of this study is the absence of a fully nourished adult control group, which limits the ability to disentangle the effects of developmental stage from prior nutritional history. Despite this, the present work provides a comprehensive framework for understanding starvation responses in *A. franciscana* and supports its utility as a model for studying metabolic stress and adaptive physiological responses.

## Figures and Tables

**Table 1:** Mortality of *Artemia franciscana* under starvation at different developmental stages.

Developmental Stage	Time (h)	Mortality (%)	*p* vs. Control	*p* vs. Nauplii (Same Time)
Nauplii	24	0	–	–
	48	1	n.s.	–
	72	73	*** *p* < 0.001	–
Adults	24	15	* *p* < 0.05	* *p* < 0.05
	48	37.5	*** *p* < 0.001	*** *p* < 0.001
	72	85	*** *p* < 0.001	n.s.

Mortality is expressed as the percentage of dead individuals relative to the total number of organisms in each group (n = 100). Statistical significance was determined using Fisher’s exact test. Differences were considered significant at * *p* < 0.05 and *** *p* < 0.001. n.s.—not significant.

## Data Availability

The original contributions presented in this study are included in the article. Further inquiries can be directed to the corresponding authors.

## Appendix A

**Table A1:** Identification of autophagy-related orthologs in crustacean species using homology-based analysis. Reference proteins representing key components of nutrient-sensing and autophagy pathways were used as queries in BLASTP searches against crustacean protein databases, with *Daphnia pulex* serving as the reference species. For each query protein, the best-matching homolog was identified and annotated, and sequence similarity metrics including query coverage, percentage identity, and E-value were recorded. The identified proteins encompass major functional modules of the canonical autophagy pathway, including nutrient sensing (*AMPK*, *TOR*, *Raptor*, *LST8*), upstream regulatory components (*FOXO*, *PTEN*, *PP2A*, *TAP46-like*), autophagy initiation (*ULK/ATG1* complex: *ATG1*, *ATG13*, *ATG101*, *RB1CC1/FIP200*), vesicle nucleation (*ATG6/Beclin-1*, *PI3K* complex), phagophore elongation (*ATG5–ATG12–ATG16* conjugation system), and *LC3/ATG8* lipidation machinery (*ATG3*, *ATG4*, *ATG7*, *ATG8*). High sequence similarity and low E-values across most identified orthologs support the evolutionary conservation of autophagy-related pathways in crustaceans. The detection of *ATG5* in a parthenogenetic lineage of Artemia further suggests lineage-specific conservation of key autophagy components. Collectively, these results provide a comprehensive overview of conserved molecular elements potentially involved in starvation responses in crustacean models.

Reference Gene	Query Species	Artemia Hit	Annotation	Query Cover	Identity	E-Value
***AMPKα***	*Daphnia pulex*	ABI13783.1	protein kinase AMPK alpha subunit 1	97%	82.14%	0.0
***TOR***	*Daphnia pulex*	XP_065560361.1	serine/threonine-protein kinase mTOR-like	100%	55.79%	0.0
***Raptor***	*Daphnia pulex*	XP_065559951.1	regulatory-associated protein of mTOR-like isoform X1	82%	56.82%	0.0
***FOXO***	*Daphnia pulex*	XP_065583871.1	forkhead box protein O-like isoform X1	86%	45.86%	3 × 10^−141^
***PTEN***	*Daphnia pulex*	XP_065567518.1	PTEN-like isoform X2	74%	44.36%	5 × 10^−116^
***ULK/ATG1***	*Daphnia pulex*	XP_065561885.1	serine/threonine-protein kinase ULK2-like isoform X2	97%	48.88%	0.0
***ATG8/LC3/GABARAP***	*Daphnia pulex*	XP_065582829.1	microtubule-associated proteins 1A/1B light chain 3C-like isoform X1	100%	39.50%	9 × 10^−27^
***ATG5 ****	*Daphnia pulex*	AKH66778.1	autophagy protein 5	98%	51.49%	1 × 10^−93^
***ATG12***	*Daphnia pulex*	XP_065579815.1	ubiquitin-like protein ATG12	67%	43.48%	6 × 10^−27^
***ATG7***	*Daphnia pulex*	XP_065581653.1	ubiquitin-like modifier-activating enzyme ATG7 isoform X3	98%	42.37%	0.0
***LST8***	*Daphnia pulex*	XP_065580346.1	target of rapamycin complex subunit lst8-like isoform X1	100%	63.04%	9 × 10^−161^
***TAP46-like***	*Daphnia pulex*	XP_065576214.1	immunoglobulin-binding protein 1-like	98%	50.00%	2 × 10^−103^
***PP2A regulatory subunit***	*Daphnia pulex*	XP_065564646.1	serine/threonine-protein phosphatase 2A 55 kDa regulatory subunit B delta isoform-like	100%	85.39%	0.0
***ATG13***	*Daphnia pulex*	XP_065556792.1	autophagy-related protein 13-like isoform X1	97%	38.73%	5 × 10^−84^
***ATG101***	*Daphnia pulex*	XP_065573695.1	autophagy-related protein 101-like	100%	58.41%	2 × 10^−97^
***RB1CC1/FIP200-like***	*Daphnia pulex*	XP_065572676.1	RB1-inducible coiled-coil protein 1-like isoform X3	73%	24.94%	2 × 10^−45^
***ATG9-like***	*Daphnia pulex*	KAK2716798.1	hypothetical protein QYM36_007070, partial	84%	47.79%	0.0
***ATG2A***	*Daphnia pulex*	XP_065564045.1	autophagy-related protein 2 homolog A-like	100%	36.32%	0.0
***WIPI2***	*Daphnia pulex*	XP_065557883.1	WD repeat domain phosphoinositide-interacting protein 2-like isoform X1	98%	60.95%	0.0
***ATG6***	*Daphnia pulex*	XP_065558699.1	beclin-1-like protein	97%	46.35%	3 × 10^−128^
***PI3K-family***	*Daphnia pulex*	XP_065568407.1	phosphatidylinositol 4,5-bisphosphate 3-kinase catalytic subunit alpha isoform-like	99%	37.20%	0.0
***PI3K regulatory subunit 4-like***	*Daphnia pulex*	XP_065557494.1	phosphoinositide 3-kinase regulatory subunit 4-like	63%	37.26%	2 × 10^−176^
***ATG16L1***	*Daphnia pulex*	XP_065576022.1	autophagy-related protein 16-1-like isoform X2	99%	47.91%	0.0
***ATG3***	*Daphnia pulex*	XP_065584499.1	ubiquitin-like-conjugating enzyme ATG3	100%	59.13%	3 × 10^−128^
***ATG4***	*Daphnia pulex*	KAK2711386.1	hypothetical protein (ATG4 homolog)	85%	46.42%	2 × 10^−109^

* detected in Artemia parthenogenetic lineage.
